# Agonist binding by the β_2_-adrenergic receptor: an effect of receptor conformation on ligand association–dissociation characteristics

**DOI:** 10.1007/s00249-015-1010-4

**Published:** 2015-03-01

**Authors:** Anita Plazinska, Wojciech Plazinski, Krzysztof Jozwiak

**Affiliations:** 1 Laboratory of Medicinal Chemistry and Neuroengineering, Department of Chemistry, Faculty of Pharmacy, Medical University of Lublin, W. Chodzki Str., 4a, 20-093 Lublin, Poland; 2 J. Haber Institute of Catalysis and Surface Chemistry, Polish Academy of Sciences, Niezapominajek Str., 8, 30-239 Cracow, Poland

**Keywords:** β_2_-Adrenergic receptor, GPCR, Ligand binding, Umbrella sampling, Free energy profiles, Molecular dynamics

## Abstract

The β_2_-adrenergic receptor (β_2_-AR), a G protein-coupled receptor (GPCR), is a physiologically important transmembrane protein that is a target for drugs used for treatment of asthma and cardiovascular diseases. Study of the first steps of ligand recognition and the molecular basis of ligand binding to the orthosteric site is essential for understanding the pharmacological properties of the receptor. In this work we investigated the characteristic features of the agonist association–dissociation process to and from the different conformational forms of β_2_-AR by use of advanced molecular modeling techniques. The investigation was focused on estimating the free energy profiles (FEPs) corresponding to the process of a full agonist ((*R*,*R*)-fenoterol) and an inverse agonist (carazolol) binding and unbinding to and from β_2_-AR. The two different conformational forms of β_2_-AR, i.e. active β_2_-AR–PDB: 3P0G and inactive β_2_-AR–PDB: 2RH1 were included in this stage of the study. We revealed several significant qualitative differences between FEPs characteristic of both conformational forms. Both FEPs suggest the existence of three transient binding sites in the extracellular domain of β_2_-AR. Comparison of the residues surrounding these transient binding sites in both β_2_-AR states revealed the importance of the aromatic residues F194, H93^2.64^, H296^6.58^, and H178 (extracellular part of β_2_-AR) in the early stages of the binding process. In addition, slightly different exit and entry paths are preferred by the ligand molecule in the extracellular part of β_2_-AR, depending on the conformation of the receptor.

**Electronic supplementary material:**

The online version of this article (doi:10.1007/s00249-015-1010-4) contains supplementary material, which is available to authorized users.

## Introduction

G protein-coupled receptors (GPCRs) are a family of seven-transmembrane receptors that, upon activation by extracellular signals, couple with trimeric G proteins or β-arrestins to transduce signals from the cellular environment into the cell. GPCRs are activated by a variety of species ranging from photons to small-molecular-weight molecules and peptides. The β_2_ adrenergic receptor (β_2_-AR) is a well-studied GPCR that mediates natural responses to the catecholamine hormones adrenaline and noradrenaline. It is crucial for physiological regulation of cardiovascular and pulmonary functions. β_2_-AR, similar to other GPCRs, consists of seven-transmembrane α-helices (TMs I–VII) connected by three extracellular (ECLs I–III) and three intracellular (ICLs I–III) loops, with an extracellular N-terminus and an intracellular C-terminus (Rosenbaum et al. 2009).

The interactions between β_2_-AR and ligands, and the mechanism of receptor activation have been intensively studied (Bai et al. 2013; Cherezov et al. 2007; Rasmussen et al. 2011a, b; Ring et al. 2013; Staus et al. 2014; Kim et al. 2013; Zocher et al. 2012; Deupi et al. 2012; Yao et al. 2006). Analysis of the ligand-binding region of β_2_-AR on the basis of recently solved high-resolution crystal structures revealed several highly conserved amino acids that might be involved in ligand binding. β_2_-AR interacts with a very diverse set of ligands which bind to the TM III, TM V, TM VI, and TM VII regions. The orthosteric binding site of β_2_-AR, which is the site of action of endogenous catecholamines, is highly conserved (Ring et al. 2013; Swaminath et al. 2005). The transmembrane part of β_2_-AR binds ligands and transduces this information to the intracellular region of the receptor that interacts with cytosolic signaling proteins. The activated GPCR stimulates the G proteins involved in the first step in the GPCR signaling cascade (Gether 2000). This event is accompanied by dynamic conformational changes in both a receptor and G proteins on a millisecond timescale. Nygaard et al. (2013) revealed the dynamic nature of GPCRs along the activation pathway by NMR experiments combined with long-term molecular dynamics (MD) simulations. Different active state conformations of GPCRs can be stabilized by different agonists, which results in their association with different downstream signaling molecules (Bokoch et al. 2010; Ghanouni et al. 2001; Kobilka and Deupi 2007; Seifert et al. 2001; Swaminath et al. 2004. Although the crystal structures of β_2_-AR provided significant insight into the structure of GPCRs and into the molecular details of interactions with different ligands, the global dynamics of the protein that lead to its activation and the functions of its extracellular sites have only been investigated more recently (Zhang et al. 2010; González et al. 2011). Apart from the involvement of the TM domains in ligand binding, it is supposed that the ECLs of β_2_-AR can also contribute to this process. In addition, the extracellular region modulates ligand access to the binding cavity. Although smaller molecules bind to the orthosteric binding site located in the TM domain, to reach the binding site they must also interact with the extracellular regions. Recent NMR studies have indicated that agonists induce conformational changes in the extracellular domain of β_2_-AR, especially in the second extracellular loop (ECL II) (Bokoch et al. 2010). Understanding the molecular basis of ligand–GPCR interactions in the extracellular surface is important, because they are implicated in ligand binding (Gkountelias et al. 2009), allosterism (Avlani et al. 2007), ligand specificity (Samson et al. 1997), and the receptor-activation process (Klco et al. 2005; Scarselli et al. 2007).

**Fig. 1:**
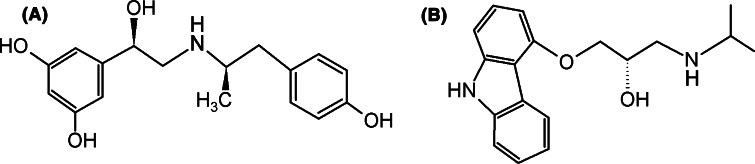
Chemical structures of (*R,R*)-fenoterol (**a**) and (*S*)-carazolol (**b**)

We have previously conducted long-term studies on the interactions of a full and selective agonist, fenoterol (Fig. 1a) and its derivatives with β_2_-AR. During these experimental studies we examined the binding of agonists (fenoterol analogs) to the different conformations of β_2_-AR, stabilized either by the agonist [^3^H](*R*,*R*)-methoxyfenoterol or by the antagonist [^3^H]CGP-12177 (Toll et al. 2012). The radioligand binding assay indicated that the agonist can bind to the active and inactive conformation of the receptor but has higher binding affinity for the β_2_-AR conformation stabilized by the agonist (active state) than for the conformation stabilized by the antagonist (inactive state). The process of antagonist displacement can affect both the orthosteric and other binding sites of β_2_-AR. Because [^3^H]CGP-12177, used as the radioligand marker, binds at two sites of the receptor (Joseph et al. 2004), it is not clear which site or sites of β_2_-AR interact with this antagonist or how these interactions affect identification and characterization of other β_2_-AR agonists. Moreover, kinetic studies performed by use of the same radioligand markers (i.e. [^3^H]-CGP-12177 and [^3^H](*R*,*R*)-methoxyfenoterol) revealed significant differences between the thermodynamic characteristics of binding of the fenoterol stereoisomers to different conformations of β_2_-AR (Toll et al. 2012). Subsequent molecular simulations confirmed experimentally determined thermodynamic data for binding (∆G^0^, ∆H^0^, ∆S^0^) and showed that structurally similar compounds (stereoisomers of fenoterol), the full agonists of β_2_-AR, bind to the inactive and active conformational states of β_2_-AR with different affinities and thermodynamic data (Jozwiak et al. 2010; Toll et al. 2012; Plazinska et al. 2014).

In these theoretical studies (docking, MD simulations) the two different high-resolution X-ray structures of β_2_-AR were used to investigate molecular interactions between β_2_-AR and fenoterol analogues. One was co-crystalized in the complex with an inverse agonist, (*S*)-carazolol (PDB: 2RH1) (Cherezov et al. 2007). This structure reveals a conserved mode of binding of antagonist and inverse agonists (Wacker et al. 2010) and is believed to be the inactive state of the receptor (Dror et al. 2011). The second structure, the active conformation, was co-crystalized with the agonist, BI-167107, and with camelid Nb80 nanobody mimicking G protein interactions with the intracellular interface of β_2_-AR (PDB: 3P0G) (Rasmussen et al. 2011a). This structure undergoes outward movement (approx 11 Å) of the cytoplasmic end of TM VI and rearrangement of TM V and TM VII, and strongly resembles that reported for opsin, an active form of a rhodopsin (Rasmussen et al. 2011a). Docking studies indicated that all of the fenoterol derivatives bind to the orthosteric site of β_2_-AR, and their pattern of the interaction is very similar to that of the BI-167107 molecule originally co-crystallized with β_2_-AR (active conformation). The 3,5-dihydroxyphenyl group of fenoterol (Fig. 1a) interacts with serines located on TM V: S203^5.42^, S204^5.43^, S20 ^5.46^ (superscript numbers correspond to the general numbering scheme of Ballesteros and Weinstein (1995)), which are involved in binding the agonist (Ring et al. 2013), inverse agonist (Cherezov et al. 2007), and antagonist (Wacker et al. 2010). The protonated amine group of the ligand creates the ionic bridge with the carboxyl group of D113^3.32^ (TM III) (Kolinski et al. 2012; Plazinska et al. 2013, 2014), by analogy with such endogenous agonist molecules as adrenaline (Ring et al. 2013). However, docking and MD simulations revealed different binding of fenoterol to the active and inactive states which result from the receptor crystal structures, especially from different distances between TM V, TM VI, and TM VII. The amine group of fenoterol can form a hydrogen bond with N312^7.39^ (TM VII) only of the active state of β_2_-AR. The fenoterol molecule does not interact simultaneously with S204, S203, S207 (TM V), and N312^7.39^ (TM VII) of the inactive conformation of β_2_-AR. Movement of TM V toward TM VI on agonist activation enables interaction between the agonist (fenoterol) and serines of TM V and N312^7.39^ (Plazinska et al. 2013). The position of the *p*-hydroxyphenyl group depends on the conformation of β_2_-AR. It can form hydrogen bonds with:

1. residues of the second extracellular loop (ECL II) (C191, D192);
2. T110^3.29^ (TM III); or
3. residues of TMVII (K305^7.32^, Y308^7.35^ or W313^7.40^).

The most significant difference between fenoterol derivatives bound to the two conformers of β_2_-AR is the hydrogen bonding contacts with K305^7.32^. Fenoterol can for hydrogen bonds with K305^7.32^ in the active conformer only. On the basis of previous computational studies we proposed that extension of the orthosteric site, located in the extracellular part of β_2_-AR and containing the TM VII (K305) and ECL II (C191, D192, F193) residues, is important in ligand binding (Plazinska et al. 2013). It has also been confirmed experimentally that Y308^7.35^ (TM VII) forms a hydrogen bond with an acceptor atom (oxygen) in the *p*-position of the fenoterol molecule (Woo et al. 2014).

Although much attention has been devoted to the nature of ligand–receptor interactions in the equilibrium structure and to the molecular mechanisms of receptor activation, the ligand association–dissociation process remains unresolved. The global dynamics of the receptor that lead to its activation and the free energy profile of ligand dissociation have been investigated more recently in computational studies (Nygaard et al. 2013; Dror et al. 2011; González et al. 2011). González et al. (2011) used the steered MD simulation to describe, in atomic detail, the process of unbinding of the two inverse agonists cyanopindolol and carazolol, which have recently been co-crystallized with the β_1_-AR and β_2_-AR subtypes. Their results indicated that cyanopindolol and carazolol gain access to the orthosteric binding site of β-AR from the extracellular environment. The forces and energies from simulation of the dissociation process also suggested the presence of intermediate binding sites located in the ECL II, ECL III, and TM VII regions, where ligands are transiently retained by electrostatic and van der Waals interactions. These binding sites were established by study of non-conserved electrostatic interactions and conserved aromatic contacts in the early stages of the binding process (González et al. 2011).

While acknowledging that the processes involved in ligand dissociation from β_2_-AR and β_1_-AR have been studied (González et al. 2011), one must note that possible differences between patterns of dissociation from the active and inactive forms of β_2_-AR are still unknown.

In this paper we describe a computational study which provides insight into agonist molecule association–dissociation with and from β_2_-AR in its active (β_2_-AR co-crystallized with the agonist, PDB: 3P0G) (Rasmussen et al. 2011a) and inactive conformational states (β_2_-AR co-crystallized with the inverse agonist (carazolol) PDB: 2RH1) (Cherezov et al. 2007). To investigate the configurational space of the receptor, and to surmount the different free energy barriers, we used the umbrella sampling (US) technique (Torrie and Valleau 1977), which enables recovery of the free energy profile (FEP) along the chosen coordinate. US has been successfully applied in studies of association–dissociation reactions of several small-molecule–protein complexes, in particular the interactions and free energy characteristics of ligand–protein complexes (Mascarenhas and Kästner 2013; Higo et al. 2012). The main objective of our study was to estimate and interpret the FEPs related to binding and unbinding of an agonist molecule ((*R*,*R*)-fenoterol), Fig. 1a, to and from β_2_-AR by taking into account that β_2_-AR can adopt distinct conformational forms (i.e. active and inactive states). The resulting FEPs were subjected to analysis revealing the main types of the ligand–receptor interaction responsible for the selected, characteristic regions of the given FEP. Next, we compared the data with results obtained for dissociation of an inverse agonist, (S)-carazolol (Fig. 1b), bound to the inactive conformer of β_2_-AR.

The system containing the agonist (fenoterol) molecule complexed with the inactive conformational state of β_2_-AR is introduced to reflect the physical process of inserting the agonist molecule into the binding cavity of β_2_-AR which is not yet activated (the inactive conformer dominates in the absence of the agonist ligand (Toll et al. 2012)). In the equilibrated β_2_-AR–agonist system the logical choice would be use of the active conformational form of the receptor which corresponds to the energy minimum of the system. This study, however, focused on association–dissociation processes far from the equilibrium states of the β_2_-AR–agonist complexes; thus, binding of fenoterol to the inactive conformational form of β_2_-AR is possible and can be regarded as one of the steps leading, eventually, to the full activation process.

## Methods

### Modeling of the ligand–receptor complexes

β_2_-AR in its inactive and active states was modeled on the basis of the crystal structure of human β_2_–AR–T4 lysozyme fusion protein (PDB: 2RH1) (Cherezov et al. 2007) and the structure of a nanobody-stabilized active state of the β_2_-AR (PDB: 3P0G) (Rasmussen et al. 2011a), respectively. A single palmitoyl chain was added to C341 at the end of the cytoplasmic helix VIII (for both structures). The Automated Topology Builder server (Canzar et al. 2013; Koziara et al. 2014) was used to obtain the ligand structures (carazolol or fenoterol) and the GROMOS force field parameters for MD simulation. The initial positions of the fenoterol molecule were determined on the basis of our previous docking study (Plazinska et al. 2013) and are in accordance with the general pattern of fenoterol–β_2_-AR interactions described in the “Introduction”. Note that the fenoterol molecules docked to the binding site of β_2_-AR interact with the same amino residues as (*S*)-carazolol and BI-167107 co-crystallized with β_2_-AR in its inactive and active conformations, respectively. (Figs. SI1 and SI2; Supporting Information). The initial position of the carazolol molecule corresponded to the crystal structure of β_2_-AR co-crystallized with carazolol (PDB: 2RH1).

The two β_2_–AR models obtained (In_β_2_-AR, representing the inactive state, and Ac_β_2_-AR, representing the active state) with bound ligands were inserted into an equilibrated palmitoyl–oleoyl–phosphatidylcholine (POPC) cell-membrane model by use of the InflateGro procedure (http://www.csb.bit.uni-bonn.de/inflategro.html) and solvated with ~16,300 simple point charge (SPC) (Berendsen et al. 1981; Van Der Spoel, et al. 1998) water molecules and two sodium ions to neutralize the total charge. Before the solvation, the ligand molecule ((*R*,*R*)-fenoterol) was inserted into the binding cavity of each of the considered proteins.

### Molecular dynamics

Energy minimization was conducted by applying 2000 steps of the steepest descent algorithm followed by 2000 steps of the l-bfgs algorithm. The four-step MD simulation then was performed with position restraints on the selected atoms (e.g. protein backbone). Finally, unconstrained MD simulation of each type of (*R,R*′)-fenoterol-β_2_-AR complexes was performed, lasting up to 100 ns. The final frames of these MD runs were used for subsequent ligand-pulling simulations (Supporting Information, Fig. SI3).

The simulations were conducted using the GROMOS 53a6 force field (Oostenbrink et al. 2004) including additional parameters for POPC molecules taken from Kukol (2009). The GROMACS 4.53 package (van Der Spoel et al. 2005) was used for all stages of MD simulations.

The PME method with a 0.9 nm cutoff (Darden et al. 1993) was used for treatment of long-range electrostatic interactions. The cutoff for Lennard–Jones interactions was 1.4 nm. These values are required for proper POPC bilayer simulation. The equations of motion were integrated by use of the leapfrog scheme (Thomas and Roe 1993) with a timestep of 2 fs. During the MD runs, the LINCS algorithm (Hess et al. 1997) was used to constrain all hydrogen atom-containing bond lengths. The simulations were performed under periodic boundary conditions based on rectangular computational boxes (initial dimensions 7.22 × 7.22 × 13.12 nm^3^). The temperature was maintained close to its reference value (310 K) by applying the V-rescale thermostat (Berendsen et al. 1984) whereas for constant pressure (1 atm, isotropic coordinate scaling) the Parrinello–Rahman barostat was used with a relaxation time of 0.4 ps (Parrinello and Rahman, 1980; 1981). Motion of the center of mass was removed every step (separately for the groups: solvent + ions, protein + ligand, lipid bilayer). The coordinates and the protein–ligand distances, and the corresponding forces (during US simulations) were saved to file every 2 ps for subsequent analysis.

### Methods of enhanced sampling

The free energy profiles (FEPs) corresponding to association–dissociation of the ligand to and from the binding cavity were calculated for the (*R,R*)-fenoterol–Ac_β_2_-AR and (*R,R*)-fenoterol–In_β_2_-AR complexes by applying the US procedure. The distance between the centers of masses of ligand and protein (*z*) was accepted as the coordinate describing the binding–unbinding process. During the first step (pulling simulations) the force was applied to the ligand center of mass to obtain the constant velocity (0.0025 nm/ps) of ligand dissociation. The vector of the force was chosen in such a way as to ensure that the ligand dissociation path will be (approximately) in accordance with the most likely path (channel C1, Fig. SI4) identified by González et al. (2011). In the next step selected frames were extracted from the resulting trajectories and accepted as starting points for the subsequent US procedure. The frames were selected to reflect the increasing value of *z* with its approximate increment of 0.1 nm and approximate initial and final values of ~2 nm (ligand fully bound) and ~5 nm (ligand outside the binding cavity). This resulted in 31 independent US simulations for each system studied. During the US runs, each lasting 40 ns, the distance between the ligand and the protein was restrained to its initial value by using the so-called umbrella potential with the force constant at 5000 kJ/mol/nm^2^. The FEP curves were calculated by using the WHAM (weighted histograms analysis method) procedure (Kumar et al. 1992) as implemented in the g_wham tool of GROMACS. The FEP-related error bars were estimated by the bootstrapping method also implemented in g_wham with the tolerance 10^−6^ and number of bins and bootstraps equal to 200 and 100, respectively.

## Results and discussion

### Analysis of the association–dissociation profiles

We start with remarks on physical interpretation of the calculated FEPs. The process studied here and reflected by the course of the free energy profiles is referred to as “association–dissociation”. The reason for this is that the US approach assumes the full equilibration of the biased MD simulations performed separately for each US window. Thus, in theory, one cannot distinguish between the association and dissociation processes on the basis of FEP expressed in terms of the accepted reaction coordinate (ligand–receptor distance), because this coordinate can describe both association and dissociation. In other words, the calculated profiles do not contain the dynamic (or kinetic) information and all the “non-equilibrium features” of the initial trajectory used to generate the US frames are assumed to be lost during 40-ns sampling.

**Fig. 2:**
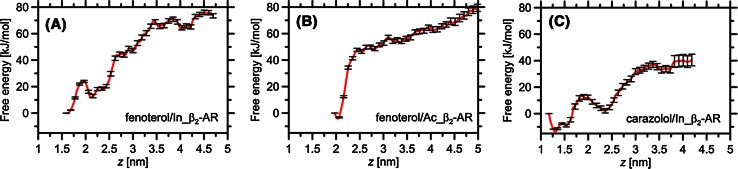
Calculated free energy profiles characteristic of the process of binding–unbinding of (*R,R*)-fenoterol and carazolol to and from the β_2_-AR binding cavity. The profiles correspond to: **a** In_β_2_-AR–carazolol, **b** In_β_2_-AR–fenoterol and **c** Ac_β_2_-AR–fenoterol complexes. *Error bars* were calculated by use of the bootstrapping method

Plots of FEP as a function of protein–ligand distance (*z*) are depicted in Fig. 2.

Differences between the global minimum (corresponding to the ligand located in the binding cavity) and the “plateau” region (corresponding to the ligand in the bulk solution, outside the receptor) for considered FEPs both were larger than those expected on the basis of experimental data (Jozwiak et al. 2010; Toll et al. 2012). Recent results (Plazinska et al. 2014) obtained for the fenoterol–β_2_-AR system which were in a good agreement with available thermodynamic data confirm that inaccuracies inherent in the force field and the initial location of the ligand were rather small. We therefore speculated that repulsive interactions between the N-terminus region and the ligand molecule (both bear a positive charge) cause the increase of the free energy. It is supposed that after dissociation of the ligand from the binding cavity, pulling it a sufficiently large distance further from the binding channel would result in a decrease of the free energy (which would then represent only ligand–solvent interactions). Although for the maximum value of the reaction coordinate the ligand is outside the binding cavity (i.e. there is no direct ligand–receptor contact) it can still interact strongly (mainly electrostatically) with the receptor, which affects the FEP values. Moving the ligand further from β_2_-AR will result in screening of the unfavorable interactions and reduction of the FEP. Consequently, the actual difference between the FEP global minimum and the characteristic FEP region for the ligand not interacting with β_2_-AR will be smaller and closer to the actual binding free energy.

Furthermore, because of the approximate course of the FEP curves, it is hard to define the exact “plateau”. The “oscillating” character of FEPs observed for larger values of the coordinate is most likely caused by interaction of the ligand with more flexible parts of the protein, e.g. the N-terminus region and extracellular loops; the timescale of this movement may be too large to enable a perfectly smooth curve to be obtained during 40 ns of sampling. (The importance of the N-terminus region is discussed in the section “Carazolol–In_β2-AR complex”). For this reason, we analyzed in detail only parts of the calculated profiles (up to *z* = 3.25–3.5 nm). In both cases the global minima can be associated with the ligand–receptor complexes studied during the previous step of the investigation (i.e. during the unconstrained MD simulations). This observation confirms the correctness of the initial structures obtained from the docking studies. First, let us notice that the minimum values of the accepted coordinate (*z*) differ for complexes involving Ac_β_2_-AR and In_β_2_-AR. This is caused both by the different conformational forms of Ac_β_2_-AR and In_β_2_-AR, which affect the optimum ligand–protein distances (expressed relative to their centers of masses) and the favorable position of the ligand, which, for Ac_β_2_-AR, is slightly shifted toward the extracellular part of the receptor; despite this shift, for both cases the spatial orientations of the ligand molecules in the binding cavity are very similar to each other. In both cases the 3,5-dihydroxyphenyl group is directed toward TM V and TM VI and the amine group of the ligand interacts with D113^3.32^ (TM III) whereas the *p*-hydroxyphenyl group is directed toward TM VII or ECL II.

For the Ac_β_2_-AR–fenoterol complex, the energy barrier between the global and first local minimum is higher by a factor of 4 (43 kJ/mol) than that observed for the In_β_2_-AR–fenoterol system (10 kJ/mol). This seems to confirm experimental studies indicating that the agonist molecule binds more strongly to β_2_-AR in its active conformation than to β_2_-AR in the inactive conformation (Toll et al. 2012). The affinity of (*R*,*R*)-fenoterol for the active conformer of β_2_-AR is 86 times higher than that for the inactive conformer (*K*_*i*.Ac_β2-AR_ = 4 nM, *K*_*i*,In_β2-AR_ = 345 nM) (Toll et al. 2012). For In_β_2_-AR, dissociation of fenoterol from the global to the local minimum of the FEP is a gradual process. In contrast with this, a sudden increase of the FEP slope at a distance between the global and first local minimum is observed for the Ac_β_2_-AR–fenoterol system. In this particular case, additional energy is required to disrupt:

1. the “ionic lock” created between the amine group of the ligand and the carboxyl group of D113^3.32^; and
2. hydrogen bonds involving, e.g., N312^7.39^ (Ac_β_2_-AR), W286^6.48^ (In_β_2_-AR), and D192, K305^7.32^ (both conformations of the receptor).

On the basis of the results obtained, we speculate that the most important difference between interaction of fenoterol with the active and inactive conformations of β_2_-AR is connected with hydrogen bonds created by the protonated amine and *β*-OH groups of the ligand and N312^7.39^. These interactions are present when the ligand is bound to active β_2_-AR but absent from the fenoterol–In_β_2_-AR complex. The above-mentioned hydrogen bonds break when the ligand leaves the favorable position in the binding cavity; this is accompanied by a shift on the FEP plot (from the global minimum to the first local minimum). This process causes the larger free energy change and the higher energy barrier between the global and first local minimum observed for the Ac_β_2_-AR conformer in comparison with the In_β_2_-AR conformer.

Below, in the section “Carazolol–In_β2-AR complex”, selected characteristic points on the FEP plots and the states corresponding to them are briefly characterized. We also describe the FEP calculated for the carazolol–In_β_2_-AR complex and compare the results with those reported elsewhere (González et al. 2011). The receptor–ligand contact maps corresponding to the selected regions of the FEP plots are shown in the Supporting Information (Figs. SI5–SI7).

#### Global minima on the FEP plots

**Fig. 3:**
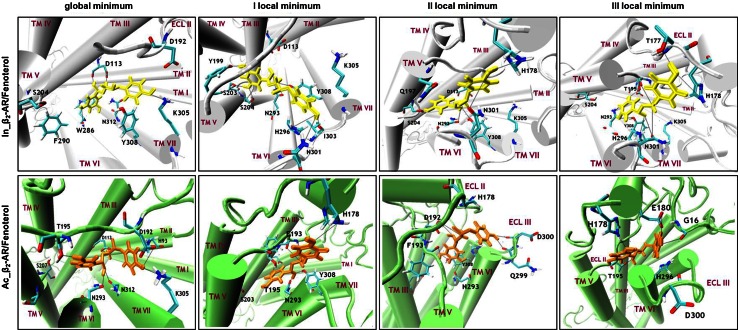
Characteristic positions (i.e. those corresponding to the local minima on the FEP curves) of the fenoterol molecule in complexes with In_β_2_-AR and Ac_β_2_-AR. The interactions are described in detail in the text and in Table 1

The global minima of both considered FEPs reflect quite different ligand–protein interaction patterns. In each case, however, the ligand position and the interaction pattern created by the characteristic groups of the ligand are conserved relative to the initial structures used in the pulling simulations. In addition, the positions of fenoterol in the global minima are very similar to the positions of the ligand co-crystallized with β_2_-AR (PDB: 2RH1 and 3P0G) (Fig. SI8). Figure 3 shows the characteristic positions of the (*R,R*)-fenoterol molecule in the binding cavities of Ac_β_2_-AR and In_β_2_-AR; both cases correspond to the global minima on the corresponding FEP plot. The main type of attractive interaction, common to both systems, is the ionic bridge between the protonated amine group of the ligand and carboxyl group of D113^3.32^. Interestingly, this interaction occurs only at the global minimum and is disrupted during further steps of the ligand-dissociation process (Table 1).

**Table 1:** Fenoterol–β_2_-AR interactions corresponding to the characteristic regions (global and local minima) of the FEP curves shown in Fig. 2

System	Ligand group	Global minimum	I Local minimum	II Local minimum	III Local minimum
In_β_2_-AR	***m*** **-OH**	W286 (backbone) **N312** **F290, F289** (Π–Π)	**Y199** T110 **S203** **S204** T195 (~4 Å)	**S203** **S204**	T195 H296
	***β*** **–OH**	**D113** Y316	**N293** **D113** **Y308** T195 (~4-5 Å)	**N293** T195 **Y308**	H296 N301 (~3–5 Å)
	–**NH**_**2**_^**+**^ **−**	**D113** T110	**Y308** **N293** T195 (~4.5 Å)	T195 **N293**	T195 (~4.5 Å)
	**CH**_**3**_	Directed toward ECL II	Directed toward ECL II	Directed toward ECL II or TM VI	F194 (hydrophobic interaction)
	***p*** **-OH**	**K305** **D192** **Y308**	I303 H296 N301 (~4–5 Å)	H178 N301 (backbone) Q197	T177 H178 (~4–8 Å)
**Ac_β**_**2**_ **-AR**	***m*** **-OH**	**S203** (~4-5 Å) T195 **F193** (backbone)	**S203** (~4–5 Å) T195 **F193, Y199** (Π–Π) **F290, F289** (Π–Π) **D113** (~4.5 Å)	**N293** (NH_2_, O) **F193** (backbone) **Y308** H296 (Π–Π)	**N293** T195 **Y308** H178, **F289** (Π–Π)
	***β*** **-OH**	**D113** **N293** **N312** **Y308**	**N293** **Y308** H296 or **F193** (backbone)	D192 **F193** (backbone)	D192 **F193** (backbone) or **Y308**
	−**NH**_**2**_^**+**^ **−**	**D113** **N312**	H296 **F193** (backbone) T195 (backbone and side chain)	D192	D192 **F193** (backbone)
	**CH**_**3**_	Toward TM III	Toward ECL II (F194)	Toward ECL II	
	***p*** **-OH**	**D192** K305 or H93	H178 F194 (Π–Π)	D300 (backbone) I298 (backbone) or H178 E180	G16 or Q179 (HB)

The interactions are separated with regard to parts of the ligand molecule (hydroxyl, amine, and methyl groups) and amino acid residues located in the vicinity of the ligand entry–exit path. “Interaction” is used here to denote close contact of the ligand molecule with the receptor resulting in an attractive interaction. If not indicated otherwise, the interactions are of the hydrogen-bonding type. Residues shown in bold are those which have been found experimentally to be involved in the interactions between the agonist and β_2_-AR (Rasmussen et al. 2011a; Ring et al. 2013; Swaminath et al. 2004; Wacker et al. 2010)

In the most favorable interaction pattern in the fenoterol–Ac_β_2_-AR complex, the amine group of the ligand is located between TM III and TM VI and can interact simultaneously with both D113^3.32^ and N312^7.39^ by salt bridge and hydrogen bonding (HB), respectively. The remaining interactions are mainly of HB-type and include:

1. HB between the 3,5-dihydroxyphenyl group of the ligand and both T195 and F193 (carbonyl oxygen atoms of the protein backbone);
2. HB between the *p*-hydroxyphenyl group (ligand) and the side chain of D192 (ECL II) or K305 (TM VII) or H93^2.64^ (TM II); and
3. HB between the *β*-hydroxyl group (*β*-OH) of the ligand and the side chain of N293^6.55^ (TM VI) or N312^7.39^.

*β*-OH–D113 hydrogen bonding is also possible but infrequent. Moreover, interaction of the ligand with D113 causes disruption of HB between *β*-OH and N293^6.55^. Lack of stable HB with the serines of TM V was observed. The distance between one of the hydroxyl groups of the 3,5-dihydroxyphenyl and S203^5.42^ is ~4–5 Å, and the contribution of the water molecule is essential for water-mediated HB with S203.

For the ligand–In_β_2_-AR complex the network of HB in the structure corresponding to the FEP global minimum is substantially different than that observed for the ligand–Ac_β_2_-AR complex. Interestingly, the ligand molecule bends relative to its main chain and the 3,5-dihydroxyphenyl group becomes closer to TM VI. The 3,5-dihydroxyphenyl group of the ligand is located between aromatic rings of W286^6.48^ and F289^6.51^ and can create very stable HB with W286 ^6.48^ (the carbonyl oxygen atom of the protein backbone). Moreover, the 3,5-dihydroxyphenyl group interacts via Π–Π stacking with the aromatic rings of W286^6.48^, F289^6.51^, F290^6.52^, F193, Y316^7.43^, and Y308^7.35^ (Fig. 3). The remaining ligand–receptor interactions characteristic of the FEP global minimum can be summarized as follows. The *p*-hydroxyphenyl group of the ligand interacts sporadically via HB with the side chains of K305^7.32^ and D192; the ligand mainly acts as an HB acceptor. Moreover, Π–Π and/or Π-hydrogen bond interactions were observed between the *p*-hydroxyphenyl group of the ligand and aromatic residues located in this area of the binding site (e.g. F193, Y308^7.35^, and F289^6.51^). Finally, the *β*-OH group participates in very stable HB with D113^3.32^ (carbonyl oxygen atom of the protein backbone); at the same time the carboxyl group of D113 forms an ionic bridge with the ligand amine group.

In both cases (i.e. for In_β_2_-AR and Ac_β_2_-AR) preferential ligand–protein interactions have their source in the strong ionic bridge created between the central part of the ligand (–NH_2_^+^ group) and the carboxyl group of D113^3.32^, accompanied by different networks of hydrogen bonds involving all the polar parts of the ligand molecule (Cherezov et al. 2007; Rasmussen et al. 2011a; Ring et al. 2013).

On comparison of the FEPs global minima obtained for In_β_2_-AR with those for Ac_β_2_-AR one can notice the lower diversity of the ligand–protein interactions in the latter case. The ligand molecule seems to be more “stable”, i.e. the accessible conformational space (expressed by the *z* value) is reduced comparing with the ligand–In_β_2_-AR complex. This is only a qualitative estimate (because of an umbrella potential bias) but it harmonizes well with one of the hypotheses explaining the diverse results of the stereoselectivity-related simulations described above (Plazinska et al. 2013). HB between *β*-OH (ligand) and N293^6.55^, which has been found to be responsible for stereoselective binding to β_2_-AR (Wieland et al. 1996), was observed for the ligand–Ac_β_2_-AR complex.

Moreover, we observed that fenoterol interacts directly (HB involving the 3,5-dihydroxyphenyl group) with W286^6.48^, i.e. the residue which creates the rotamer toggle switch, and is involved in the process of receptor activation. This type of interaction has not previously been observed in any agonist–β_2_-AR system; we were also unable to observe it during unbiased MD simulations, which suggests that such interactions are artifacts inherent in the (biased) US simulation. This issue will be discussed in detail in forthcoming papers.

#### Fenoterol–Ac_β_2_-AR complex: local minima on the FEP plot

Extraction of fenoterol through the channel in β_2_-AR reveals the existence of multiple retention points represented by the local minima on the FEP plots. The most significant of these are located at approximately *z* = 2.5 nm (Ac_β_2_-AR) and *z* = 2.15 nm (In_β_2_-AR) (Fig. 2). These “secondary” binding sites and the ligand–receptor interactions corresponding to them are briefly characterized below, starting from Ac_β_2_-AR.

For the ligand–Ac_β_2_-AR complex the first local minimum (*z* = 2.5 nm) of the FEP is determined mainly by ligand–receptor HB. Table 1 lists the protein residues located closest to the ligand during its dissociation process. The largest free energy differences between the global and first local minimum arise as a result of:

1. the lack of an ionic bridge between the protonated amine group of fenoterol and D113^3.32^ and the lack of any direct ligand–protein interactions engaging the ligand amine group (except the extremely scarce HB with F193) in general; and
2. the slightly changed position of the *p*-hydroxyphenyl group which can interact with D192 and H178 (ECL II), but not simultaneously.

The remaining (HB-type) interactions are comparable for both the global minimum and first local minimum, leading to the conclusion that disruption of the ligand–D113 salt bridge is responsible for the large increase on the FEP plot for *z* increments varying from 2 to 2.5 nm, corresponding to the transition from the main to the secondary minimum of free energy.

Furthermore, the additional (very shallow) local minimum on the FEP curve is visible at approximately *z* = 2.78 nm, as a result of four ligand–receptor interactions:

1. strong HB between the *p*-hydroxyphenyl group (ligand) and the side chain of D300 and Q299 (the same *p*-hydroxyphenyl group can also interact (but less strongly) with H178 located on the ECL II);
2. HB of the dihydroxyphenyl group of fenoterol with N293^6.55^, Y308^7.35^, and F193 (less frequent HB involving H296^6.58^ is also observed);
3. during exit from the first local minimum, HB between the ligand *β*-OH group and N312 is lost, resulting in rotation of the β-OH and directing it towards the extracellular part of β_2_-AR; at the second local minimum the β-OH group interacts via HB with D192 (frequently) and F193 (backbone atoms, infrequently) but not simultaneously; and
4. the amine group (ligand) acts as an HB donor to D192.

**Fig. 4:**
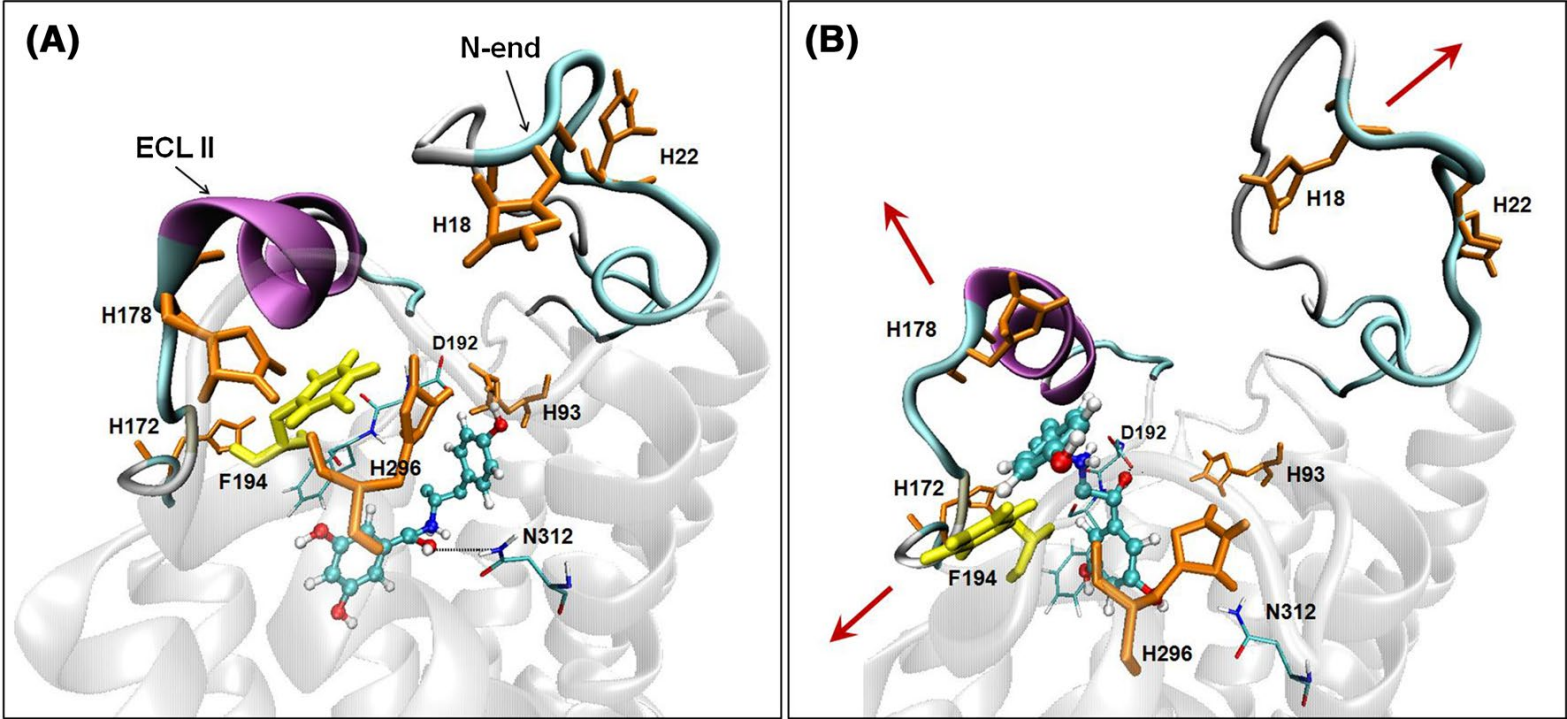
The (*R*,*R*)-fenoterol molecule (ball-and-stick representation) leaving the first local minimum (**a**) and entering the second local minimum (**b**) of FEP. *Red arrows* show the directions of ECL II and N-end movements which accompany the ligand transfer

The ligand molecule has a bent shape with the–NH_2_^+^– group exposed to the solvent environment. On the basis of observation of the MD trajectories representing ligand transfer from the first to the second local minimum of FEP, we suggest a novel effect of F194 (ECL II) and histidines: H93^2.64^, H296^6.58^, and H178. These histidines, surrounding the F194 side chain, can create a network of Π–Π interactions with each other (Fig. 4a), with F194, and with the aromatic group of the ligand. We hypothesize that in the process of ligand entry and exit to and from the binding cavity the network of interactions involving H93^2.64^, H296^6.58^, F194, and H178 (ECL II) is disrupted, which results in the shift of ECL II and the N-end and, at the same time, increases the space available for the ligand molecule. This enables the *β*-OH and NH_2_^+^ groups to participate in stable interactions with D192 and F193 (ECL II) (Fig. 4b).

The course of the FEP plot reveals the existence of the third local minimum for the ligand complex with Ac_β_2_-AR at approximately *z* = 3.3 nm. There the agonist molecule interacts mainly with the residues of ECL II. The interactions with N293^6.55^, D192, F193, and T195 are still observed (Fig. 3). Moreover, an HB-type interaction between the *p*-hydroxyphenyl group (ligand) and G16 (N-end) was observed. The latter type of interaction is especially interesting because of the β_2_-AR polymorphism at the 16th residue; it will be studied in more detail in forthcoming studies.

#### Fenoterol–In_β_2_-AR complex: local minima on the FEP plot

For both local and global minima of the free energy only very few types of ligand–protein interaction can be distinguished for In_β_2_-AR. All are very stable, however. The first and the second local minima of the free energy are located very close to each other, at approximately *z* = 1.7 nm and *z* = 2.1 nm, respectively. They are separated by a relatively small free energy barrier (~9 kJ/mol). The orientations of the fenoterol molecule corresponding to these two minima are quite similar. The slight differences are mainly because of orientation of the *p*-hydroxyphenyl group. At the first local minimum, the *p*-hydroxyphenyl group is closer to ECL III whereas at the second local minimum it is shifted toward ECL II (Fig. 3).

The attractive interactions associated with the first local minima of the free energy are limited to:

1. simultaneous HB between the two hydroxyl groups of the 3,5-dihydroxyphenyl ligand group and two of the three residues S203^5.42^, S204^5.43^, and Y199;
2. the *β*-OH group of fenoterol interacts directly (HB) with N293^6.55^, which has previously been identified as responsible for stereoselective binding to the receptor (Wieland et al. 1996);
3. the protonated amine group donates a hydrogen bond to Y308^7.35^ and N293^6.55^; and
4. the *p*-hydroxyphenyl group participates in HB involving I303, H296^6.58^, or N301.

Similarly to Ac_β_2_-AR, in this case also, disruption of the ligand–D113 salt bridge can be interpreted as the main reason for the increase of the free energy at *z* distances varying from 1.7 to 2.1 nm (i.e. the transition of the ligand molecule from the main free energy minimum to the second).

Most of the mentioned interactions involving the 3,5-dihydroxyphenyl, *β*-OH, and amine groups of the ligand are also maintained for the configurations representing the second local minimum of the free energy (*z* = 2.35 nm; Figs. 2, 3). The *p*-hydroxyphenyl group, located between TM V, ECL II, and ECL III in the extracellular part of the receptor, participates in HB with H178 (ECL II), Q197^5.36^ (TM V), or N301 (ECL III). In contrast with the fenoterol–Ac_β_2_-AR complex, stable interactions with serines on TM V were observed for both the first and second minima of FEP. It is supposed that these attractive interactions partially compensate for the energetically unfavorable disruption of the D113-ligand salt bridge and are the reason for the relatively small (16–19 kJ/mol) FEP difference between the global and the first two local minima characteristic of In_β_2_-AR. For Ac_β_2_-AR, in which interactions with serines are absent, the corresponding difference is much larger (45 kJ/mol).

The third local minimum (*z* = 2.8 nm) corresponds to the ligand shifted closer to the extracellular part of the receptor. The fenoterol molecule loses its direct interactions with the TM V (S204), TM VI (N293), and TM VII (Y308) residues. The amine group of the ligand is located between ECL II and ECL III and does not participate in HB with β_2_-AR. Overall, the ligand molecule interacts only with residues on the extracellular loops. The 3,5-dihydroxyphenyl group participates in HB simultaneously with T195 (ECL II) and H296^6.58^. The *β*-OH group forms two hydrogen bonds, with H296^6.58^ and N301 (ECL III). The *p*-hydroxyphenyl group of fenoterol can interact with the T177 residue (ECL II) only. The stability of these HBs is weakened by the large mobility of the extracellular part of β_2_-AR.

#### Carazolol–In_β_2_-AR complex

In contrast with the fenoterol–β_2_-AR complexes, the FEP plot obtained for carazolol–In-_β_2_-AR reveals the existence of the global minimum (*z* = 1.25) and only two local minima (*z* = 1.5, *z* = 2.32 nm). The ligand position in the global minimum is very similar to that observed in the crystal structure of the carazolol–β_2_-AR complex (PDB: 2RH1); the average RMSD was 0.21 Å (Fig. SI5).

The most favorable interactions in the carazolol–In_β_2_-AR complex are created between the amine group of the ligand and D113^3.32^, N312^7.39^, and Y316^7.43^. The other interactions include HB between:

1. serines on the TM V (S203^5.42^, S204^5.43^) and the aromatic groups of ligand; and
2. the hydroxyl group (ligand) and D113^3.32^ (Table 2).obtained from

**Fig. 5:**
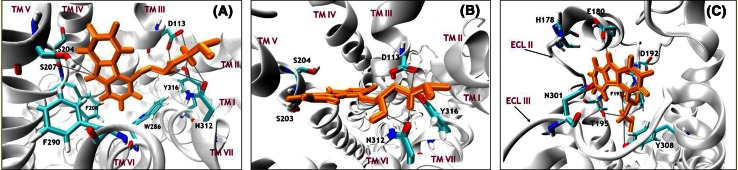
Schematic diagram of interactions between the carazolol molecule and In_β_2_-AR in the global minimum (**a**), 1st local minimum (**b**), and 2nd local minimum (**c**) of FEP

In contrast with the fenoterol–β_2_-AR complexes, disruption of HB between the hydroxyl group of carazolol and D113 seems to be of fundamental importance for dissociation from the global minimum. Slight rotation of the ligand β-OH group causes disruption of the *β*-OH–D113 HB and creation of new HB between *β*-OH and N312^7.39^. Moreover, the ligand becomes more distant from the serines on TM V. The distances between the nitrogen atom of the aromatic ring (ligand) and S203^5.42^ and S204^5.43^ increases to 4–6 Å (Table 2). This change in the interaction pattern affects the FEP values and the distance between the global and 1st local minimum to a relatively minor extent. In the first local minimum the protonated amine group of ligand still interacts with D113^3.32^, N312^7.39^, and Y316^7.43^. Stronger interactions with residues on TM III (D113) and TM VII (N312), compared with fenoterol, seem to be the result of the different chemical structures of the ligands. In the carazolol molecule the distance between the aromatic and amine groups is larger by two C–C bonds than for the fenoterol molecule; this enables the carazolol molecule to maintain stable interactions with N312^7.39^ without disrupting the network of interactions around the aromatic group. A significant energy barrier located between the first and second local minima is associated with disruption of HB between the ligand and the D113^3.32^, N312^7.39^, and Y316^7.43^ residues (Fig. 5, Table 2).

**Table 2:** The β_2_-AR amino acid residues identified as interacting with carazolol during its association–dissociation process

Ligand group	Global minimum	I Local minimum	II Local minimum
Aromatic rings	**S203** **S204** **S207** **V114, F193 (П-П)** **F289, F290, W286 (П-П)**	**S204** **S203** **F289, F290 (П-П)** **F193, V114 (П-П)**	N301 E180 H178 (distance 0.4 nm) or H18
–OH	**D113**	**N312** **N293**	T195 **F193** Y308 **D192**
–NH_2_^+^–	**D113** **Y316** **N312**	**D113** **N312**	T195 **F193** Y308
CH_3_	**W109** V86 G90	**H93** **W109** I309	**F193** I309

Residues shown in bold are those which have been found experimentally to be involved in the interactions between inverse agonist and β_2_-AR (Cherezov et al. 2007, Wacker et al. 2010). Other details as in Table 1

It is worth briefly comparing these results with those obtained by González et al. (2011) for the β_2_-AR–cyanopindolol and β_2_-AR–carazolol systems. The authors used the Jarzynski equality and pulling simulations to recover the FEP associated with the ligand (carazolol) dissociation processes. For both the fenoterol–β_2_-AR and carazolol–β_2_-AR complexes, the residues involved in direct contact with the ligand are similar to those reported by González et al. (2011). In contrast with González et al. (2011) we observed metastable states characteristic of the ligand-dissociation process (local minima of the free energy).

The differences between the FEP profiles calculated for fenoterol and carazolol could result from three factors:

1. The simulation techniques used which, despite seeking the same quantity (FEP), can lead to slightly different results because of accuracy-related issues. The use of steered MD and Jarzynski equality assumes the ligand dissociation process (as accepted by the pulling direction) whereas use of time-independent umbrella potentials results in ligand association and dissociation processes described by the same profile (reversibility is assumed). The sampling times (~30 ns for each US window) are much larger than the time of ligand pulling (~3 ns) described by González et al. (2011), allowing the assumption that the conformational space is sampled more extensively in the US simulations.
2. The types of the ligands, which have two different pharmacological characteristics (agonist and inverse agonist); moreover, the dimensions of their molecules differ substantially (the structural formulas are given in Fig. 1). For steric reasons it was expected that the larger fenoterol molecule would experience different (probably larger) free energy barriers than carazolol when entering and leaving the binding cavity. The importance of ligand physiological character is confirmed by the metadynamics simulations (described below).
3. Details of the composition of the systems. The FEP profiles reported by González et al. (2011) were obtained on the basis of the crystal structure of β_2_-AR (PDB: 2RH1) which did not contain the uncrystallized region of the N-terminus which, according to our US simulations, can be of crucial importance in the initial stages of the ligand-binding process. The proximity of the positively charged N-teminus region interacting with the cationic ligand can explain the larger free energy barrier observed for *z* > 3 nm compared with the equivalent results obtained for carazolol. The artificial N-terminus of the incomplete crystal structure of β_2_-AR would be located in the vicinity of the extracellular loops and obstructs entry to the core and/or binding site of β_2_-AR, i.e. further from the preferred ligand dissociation path. The fully reconstructed N-terminus region has much conformational flexibility which has a larger effect on the ligand–β_2_-AR interaction pattern.

### Ligand exit and entry paths

To summarize, the most significant differences between the ligand binding–unbinding modes characteristic of the Ac_β_2_-AR and In_β_2_-AR conformational forms include:

1. the ligand positions in the global and local minima of the free energy;
2. the quantitatively different course of the FEP plots associated with the binding–unbinding processes; and
3. the favorable entry and exit routes of the ligand to and from the binding cavity of Ac_β_2_-AR and In_β_2_-AR (described below).

**Fig. 6:**
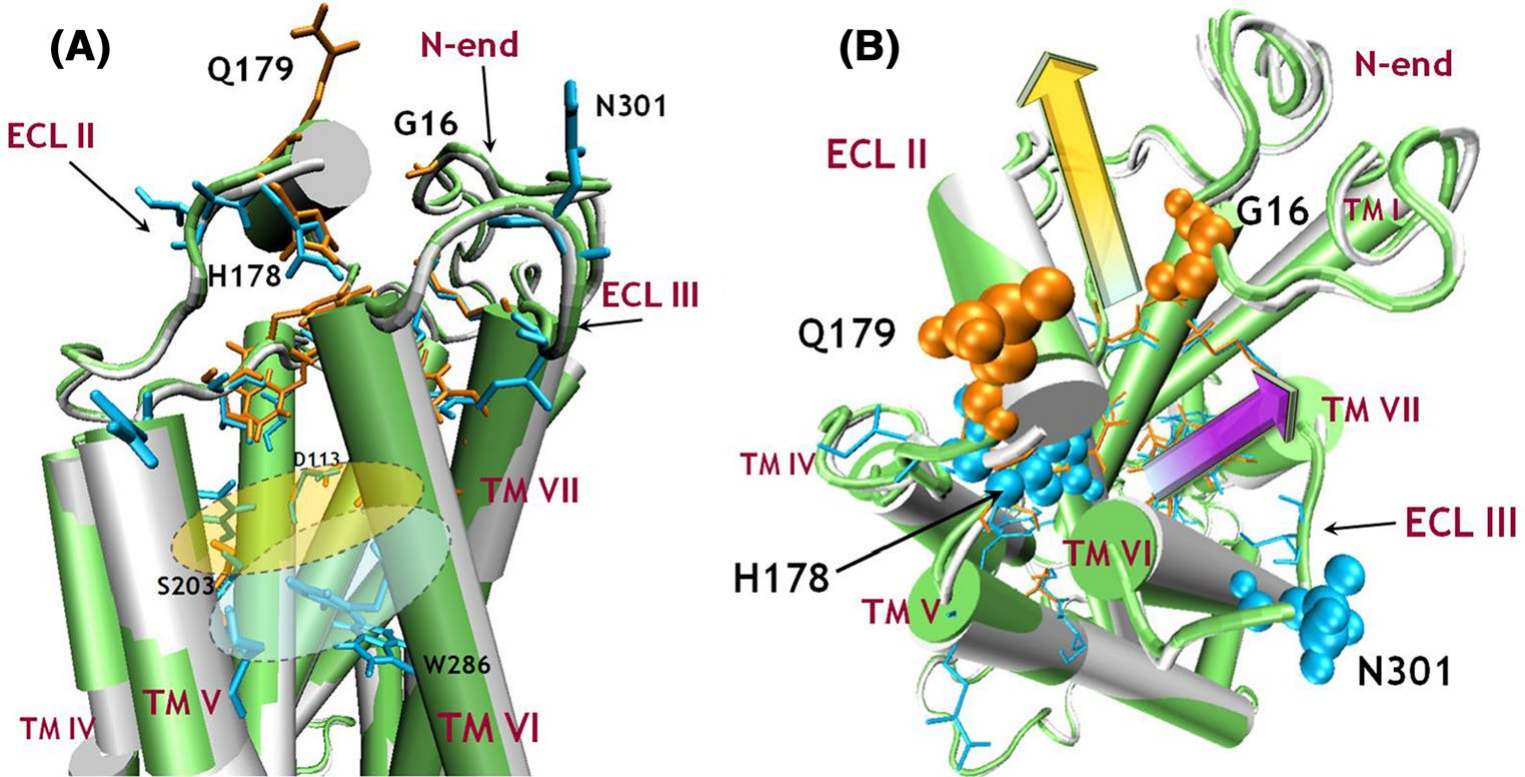
**a** Schematic depiction of the slightly different ligand entry and exit paths found for Ac_β_2_-AR and In_β_2_-AR, marked as *yellow* and *purple* *arrows*, respectively (**b**). The optimum ligand positions characteristic of Ac_β_2_-AR and In_β_2_-AR are symbolically denoted as *yellow* and *grey ellipses*, respectively. The balls denote the residues (*orange*, Ac_β_2_-AR; *blue*, In_β_2_-AR) along the extraction trajectories and belonging to ECL II, ECL III, and the N-end, which directly interact with the fenoterol molecule

When considering the global minimum of FEP, we observed that the ligand molecule in the fenoterol–Ac_β_2_-AR complex is closer to the extracellular part of the receptor (Fig. 6, yellow ellipse) than in the In_β_2_-AR complex. In the latter, the ligand is situated deeper in the cavity and can interact directly with W286^6.48^ (Fig. 6, gray ellipse).

As expected on the basis of different ligand–receptor interaction patterns (described above), slightly different dissociation paths of the ligand from Ac_β_2_-AR and In_β_2_-AR were also observed, diverging especially in the extracellular part of β_2_-AR. This determines the different pattern of ligand–receptor interactions in the third local minimum of the free energy (Fig. 3). During the whole dissociation process from In_β_2_-AR the ligand molecules, (*R*,*R*)-fenoterol and carazolol, were closer to TM V in comparison with Ac_β_2_-AR. Fenoterol interacts with S203^5.42^ and S204^5.43^ only when the system state corresponds to the first and second local minima of free energy (for In_β_2_-AR).

Moreover, for the third local minimum (In_β_2_-AR) the protonated amine group of fenoterol interacts with T195 (ECL II), being much closer to this residue than in the fenoterol–Ac_β_2_-AR complex (Fig. 3). With Ac_β_2_-AR, fenoterol leaves the third local free energy minimum and exits the binding pocket interacting with residues belonging to ECL II and the N-end (orange arrow, Fig. 6). The analogous scenario for the fenoterol–In_β_2_-AR complex is with different, because the ligand exits the binding cavity of receptor between ECL II and ECL III (purple arrow, Fig. 6). It should be strongly emphasized that the different paths were not imposed during the initial pulling simulation; the pulling directions were identical in both cases and the path divergence has its source in the specific ligand–receptor interactions characteristic of both conformational forms of β_2_-AR. We hypothesize that the increasing distance between D192 and K305^7.32^ and disruption of Π–Π interactions between the *p*-hydroxyphenyl group of the ligand, F194, and histidines (H18, H93^2.64^, H178, H296^6.58^) creates the additional space for the ligand extracted from Ac_β_2_-AR (Fig. 4) when fenoterol interacts more strongly with D192 than with In_β_2_-AR (Table 1). Finally, only for the fenoterol–Ac_β_2_-AR complex was direct interaction of the ligand with G16 observed. The experimental studies suggest that the β_2_-AR Arg/Gly16 polymorphism may be an important genetic factor in the overall risk of developing asthma (Xie et al. 2014).

Importantly, all the TM residues identified in our study, located at positions 2.64 (H93 in β_2_-AR) (Xie et al. 2006), 3.28 (W109 in β_2_-AR) (Cherezov et al. 2007; Gerber et al. 2001; Hogan et al. 2006), 3.32 (D113 in β_2_-AR) (Cherezov et al. 2007; Rasmussen et al. 2011a; Ring et al. 2013), 5.36 (Q197 in β_2_-AR) (Gether et al. 1994), 5.42, (S203 in β_2_-AR), 5.43 (S204 in β_2_-AR), 5.46 (S207 in β_2_-AR) (Cherezov et al. 2007; Cummings et al. 2010; Del Carmine et al. 2002; Rasmussen et al. 2011a; Ring et al. 2013), 6.48, 6.51, 6.52 (W286, F289, F290 in β_2_-AR) (Cherezov et al. 2007), 6.55 (N293 in β_2_-AR) (Wieland et al. 1996), 6.58 (H296 in β_2_-AR) (Hovelmann et al. 2002), 7.35 (Y308 in β_2_-AR) (Woo et al. 2014), 7.36 (I309 in β_2_-AR) (Jarnagin et al. 1996), 7.39 (N312 in β_2_-AR) (Cherezov et al. 2007; Suryanarayana and Kobilka 1993), and 7.40 (W313 in β_2_-AR) (Roth et al. 1997) have been experimentally confirmed to be involved in ligand binding to β-AR and/or other GPCRs.

## Summary

Enhanced sampling MD simulations have been performed to calculate the free energy profiles associated with the full agonist ((*R*,*R*)-fenoterol) and inverse agonist (carazolol) association–dissociation process to and from the β_2_-adrenergic receptor (β_2_-AR). For fenoterol, the calculations were performed separately for the two conformational forms of β_2_-AR, i.e. the inverse agonist-bound (inactive form, PDB: 2RH1) and the agonist-bound (active form, PDB 3POG) to elucidate potential similarities and differences.

For both conformational forms of β_2_-AR binding of the fenoterol ligand is a highly favorable process, whereas its unbinding requires overcoming of large (tens of kJ/mol) free energy barriers. The difference between the global minima and “plateaus” reach 80 kJ/mol for complexes containing fenoterol and 50 kJ/mol for carazolol. Note that these values do not necessarily correspond to the free energy of binding, as explained in the section “Analysis of the association–dissociation profiles”. Despite the number of similarities, the free energy profile calculated for the inactive form of the receptor has more “rough” character than that characteristic of the active β_2_-AR, with more metastable states and hindrance points experienced by the ligand during its binding and unbinding. Analysis of the profiles enabled identification of the crucial type of interactions responsible for each characteristic region of the free energy profiles. For both forms of β_2_-AR leaving the primary global minimum of the free energy is connected with disruption of the most favorable, attractive ligand–receptor interaction, i.e. disruption of the salt bridge between D113^3.32^ and the positively charged amine group of the ligand. For the carazolol–β_2_-AR complex, leaving the global minimum is associated with disruption of HB between the hydroxyl group of the ligand and the carboxyl group of D113. Irrespective of the conformational form of β_2_-AR, most of the attractive fenoterol–receptor interactions in the secondary minima of the free energy (representing intermediate, metastable states of the ligand undergoing the binding–unbinding process) are of hydrogen-bonding-type. Further differences between the active and inactive states of β_2_-AR include the slightly different association–dissociation paths of the ligand in the extracellular parts of the receptor. Comparison of these results with those reported for the inverse agonist (carazolol) molecule binding to the inactive form of β_2_-AR led to speculation about the effect of the N-termini in the initial stages of ligand-binding process. Finally, we observed that the fenoterol molecule may interact directly with the W286^6.48^ residue when is bound by the inactive β_2_-AR conformer. This observation can lead to better understanding of the effect of the ligand on the initial steps of the β_2_-AR conformational rearrangements (“activation”) but requires further studies.

## Electronic supplementary material

Below is the link to the electronic supplementary material.

Supplementary material 1 (DOCX 3153 kb)

## References

[CR1] Avlani VA, Gregory KJ, Morton CJ, Parker MW, Sexton PM (2007). Critical role for the second extracellular loop in the binding of both orthosteric and allosteric G protein-coupled receptor ligands. J Biol Chem.

[CR2] Bai Q, Zhang Y, Ban Y, Liu H, Yao X (2013). Computational study on the different ligands induced conformation change of β2 adrenergic receptor-Gs protein complex. PLoS one.

[CR3] Ballesteros JA, Weinstein H (1995). Integrated methods for the construction of three dimensional models and computational probing of structure-function relations in G-protein coupled receptors. Methods in Neurosciences.

[CR4] Berendsen HJC, Postma JPM, van Gunsteren WF, Hermans J, Pullman B (1981). Interaction models for water in relation to protein hydration. Intermolecular Forces.

[CR5] Berendsen HJC, Postma JPM, van Gunsteren WF, DiNola A, Haak JR (1984). Molecular dynamics with coupling to an external bath. J Chem Phys.

[CR6] Bokoch MP, Zou Y, Rasmussen SG, Liu CW, Nygaard R (2010). Ligand-specific regulation of the extracellular surface of a G-protein-coupled receptor. Nature.

[CR7] Canzar S, El-Kebir M, Pool R, Elbassioni K, Malde AK, Mark AE, Geerke DP, Stougie L, Klau GW (2013). Charge group partitioning in biomolecular simulation. J Comput Biol.

[CR8] Cherezov V, Rosenbaum DM, Hanson MA, Rasmussen SG, Thian FS, Kobilka TS, Choi HJ, Kuhn P, Weis WI, Kobilka BK, Stevens RC (2007). High-resolution crystal structure of an engineered human beta2-adrenergic G protein-coupled receptor. Science.

[CR9] Cummings DF, Ericksen SS, Goetz A, Schetz JA (2010). Transmembrane segment five serines of the D4 dopamine receptor uniquely influence the interactions of dopamine, norepinephrine, and Ro10-4548. J Pharmacol Exp Ther.

[CR10] Darden T, York D, Pedersen L (1993). Particle mesh Ewald: an N•log (N) method for Ewald sums in large systems. J Chem Phys.

[CR11] Del Carmine R, Ambrosio C, Sbraccia M, Cotecchia S, Ijzerman AP, Costa T (2002). Mutations inducing divergent shifts of constitutive activity reveal different modes of binding among catecholamine analogues to the beta(2)-adrenergic receptor. Br J Pharmacol.

[CR12] Deupi X, Li X-D, Schertler GFX (2012). Ligands stabilize specific GPCR conformations: but how?. Structure.

[CR13] Dror RO, Arlow DH, Maragakis P, Mildorf TJ, Pan AC, Xu H, Borhani DW, Shaw DE (2011). Activation mechanism of the β_2_-adrenergic receptor. PNAS.

[CR14] Gerber BO, Meng EC, Dotsch V, Baranski TJ, Bourne HR (2001). An activation switch in the ligand binding pocket of the C5a receptor. J Biol Chem.

[CR15] Gether U (2000). Uncovering molecular mechanisms involved in activation of G protein-coupled receptors. Endocr Rev.

[CR16] Gether U, Nilsson L, Lowe JA, Schwartz TW (1994). Specific residues at the top of transmembrane segment V and VI of the neurokinin-1 receptor involved in binding of the nonpeptide antagonist CP 96,345. J Biol Chem.

[CR17] Ghanouni P, Gryczynski Z, Steenhuis JJ, Lee TW, Farrens DL (2001). Functionally different agonists induce distinct conformations in the G protein coupling domain of the beta 2 adrenergic receptor. J Biol Chem.

[CR18] Gkountelias K, Tselios T, Venihaki M, Deraos G, Lazaridis I (2009). Alanine scanning mutagenesis of the second extracellular loop of type 1 corticotropin-releasing factor receptor revealed residues critical for peptide binding. Mol Pharmacol.

[CR19] González A, Perez-Acle T, Pardo L, Deupi X (2011). Molecular Basis of Ligand Dissociation in β-Adrenergic Receptors. PLoS one.

[CR20] Hess B, Bekker H, Berendsen HJC, Fraaije JGEM (1997). LINCS: a linear constraint solver for molecular simulations. J Comp Chem.

[CR21] Higo J, Ikebe J, Kamiya N, Nakamura H (2012). Enhanced and effective conformational sampling of protein molecular systems for their free energy landscapes. Biophys Rev.

[CR22] Hogan K, Peluso S, Gould S, Parsons I, Ryan D (2006). Mapping the binding site of melanocortin 4 receptor agonists: a hydrophobic pocket formed by I3.28 (125), I3.32 (129), and I7.42 (291) is critical for receptor activation. J Med Chem.

[CR23] Hovelmann S, Hoffmann SH, Kuhne R, ter Laak T, Reilander H (2002). Impact of aromatic residues within transmembrane helix 6 of the human gonadotropin-releasing hormone receptor upon agonist and antagonist binding. Biochemistry.

[CR24] Jarnagin K, Bhakta S, Zuppan P, Yee C, Ho T (1996). Mutations in the B2 bradykinin receptor reveal a different pattern of contacts for peptidic agonists and peptidic antagonists. J Biol Chem.

[CR25] Joseph SS, Lynham JA, Colledge WH, Kaumann AJ (2004). Binding of (−)-[3H]-CGP12177 at two sites in recombinant human beta 1-adrenoceptors and interaction with beta-blockers. Naunyn Schmiedebergs Arch Pharmacol.

[CR26] Jozwiak K, Toll L, Jimenez L, Woo AY, Xiao RP, Wainer IW (2010). The effect of stereochemistry on the thermodynamic characteristics of the binding of fenoterol stereoisomers to the beta(2)-adrenoceptor. Biochem Pharmacol.

[CR27] Kim TH, Chung KY, Manglik A, Hansen AL, Dror RO, Mildorf TJ, Shaw DE, Kobilka BK, Prosser RS (2013). The role of ligands on the equilibria between functional states of a G protein-coupled receptor. J Am Chem Soc.

[CR28] Klco JM, Wiegand CB, Narzinski K, Baranski TJ (2005). Essential role for the second extracellular loop in C5a receptor activation. Nat Struct Mol Biol.

[CR29] Kobilka BK, Deupi X (2007). Conformational complexity of G-protein-coupled receptors. Trends Pharmacol Sci.

[CR30] Kolinski M, Plazinska A, Jozwiak K (2012). Recent progress in understanding of structure, ligand interactions and the mechanism of activation of the β_2_-adrenergic receptor. Curr Med Chem.

[CR31] Koziara KB, Stroet M, Malde AK, Mark AE (2014). Testing and validation of the Automated Topology Builder (ATB) version 2.0: prediction of hydration free enthalpies. Journal of Computer-Aided Molecular Design.

[CR32] Kroeze WK, Sheffler DJ, Roth BL (2003). G-protein-coupled receptors at a glance. J Cell Sci.

[CR33] Kukol A (2009). Lipid models for united-atom molecular dynamics simulations of proteins. J Chem Theory Comput.

[CR34] Kumar S, Bouzida D, Swendsen RH, Kollman PA, Rosenberg JM (1992). The weighted histogram analysis method for free-energy calculations on biomolecules. I. The method. J Comput Chem.

[CR35] Mascarenhas NM, Kästner J (2013). How maltose influences structural changes to bind to maltose-binding protein: results from umbrella sampling simulation. Proteins.

[CR36] Nygaard R, Zou Y, Dror RO, Mildorf TJ, Arlow DH, Manglik A, Pan AC, Liu CW, Fung JJ, Bokoch MP, Thian FS, Kobilka TS, Shaw DE, Mueller L, Prosser RS, Kobilka BK (2013). The dynamic process of β2-adrenergic receptor activation. Cell.

[CR37] Oostenbrink C, Villa A, Mark AE, van Gunsteren WF (2004). A biomolecular force field based on the free enthalpy of hydration and solvation: the GROMOS force-field parameter sets 53A5 and 53A6. J Comput Chem.

[CR38] Parrinello M, Rahman A (1980). Crystal structure and pair potentials: a molecular dynamics study. Phys Rev Lett.

[CR39] Parrinello M, Rahman A (1981). Polymorphic transitions in single crystals—a new molecular dynamics method. J Appl Phys.

[CR40] Plazinska A, Kolinski M, Wainer IW, Jozwiak K (2013). Molecular interactions betweenfenoterol stereoisomers and derivatives and the β2-adrenergic receptor binding site studied by docking and molecular dynamics simulations. J Mol Model.

[CR41] Plazinska A, Plazinski W, Jozwiak K (2014). Fast, metadynamics-based method for prediction of the stereochemistry-dependent relative free energies of ligand–receptor interactions. J Comp Chem.

[CR42] Rasmussen SG, Choi HJ, Fung JJ, Pardon E, Casarosa P, Chae PS, Devree BT, Rosenbaum DM, Thian FS, Kobilka TS, Schnapp A, Konetzki I, Sunahara RK, Gellman SH, Pautsch A, Steyaert J, Weis WI, Kobilka BK (2011). Structure of a nanobody-stabilized active state of the β(2) adrenoceptor. Nature.

[CR43] Rasmussen SG, DeVree BT, Zou Y, Kruse AC, Chung KY, Kobilka TS, Thian FS, Chae PS, Pardon E, Calinski D, Mathiesen JM, Shah ST, Lyons JA, Caffrey M, Gellman SH, Steyaert J, Skiniotis G, Weis WI, Sunahara RK, Kobilka BK (2011). Crystal structure of the β_2_ adrenergic receptor-Gs protein complex. Nature.

[CR44] Ring AM, Manglik A, Kruse AC, Enos MD, Weis WI, Garcia KC, Kobilka BK (2013). Adrenaline-activated structure of β_2_-adrenoceptor stabilized by an engineered nanobody. Nature.

[CR45] Rosenbaum DM, Rasmussen SG, Kobilka BK (2009). The structure and function of G-protein-coupled receptors. Nature.

[CR46] Roth BL, Shoham M, Choudhary MS, Khan N (1997). Identification of conserved aromatic residues essential for agonist binding and second messenger production at 5-hydroxytryptamine2A receptors. Mol Pharmacol.

[CR47] Samson M, LaRosa G, Libert F, Paindavoine P, Detheux M (1997). The second extracellular loop of CCR5 is the major determinant of ligand specificity. J Biol Chem.

[CR48] Scarselli M, Li B, Kim SK, Wess J (2007). Multiple residues in the second extracellular loop are critical for M3 muscarinic acetylcholine receptor activation. J Biol Chem.

[CR49] Seifert R, Wenzel-Seifert K, Gether U, Kobilka BK (2001). Functional differences between full and partial agonists: evidence for ligand-specific receptor conformations. J Pharmacol Exp Ther.

[CR50] Staus DP, Wingler LM, Strachan RT, Rasmussen SG, Pardon E, Ahn S, Steyaert J, Kobilka BK, Lefkowitz RJ (2014). Regulation of beta-2-adrenergic receptor function by conformationally selective single-domain intrabodies. Mol Pharmacol.

[CR51] Suryanarayana S, Kobilka BK (1993). Amino acid substitutions at position 312 in the seventh hydrophobic segment of the beta2-adrenergic receptor modify ligand-binding specificity. Mol Pharmacol.

[CR52] Swaminath G, Xiang Y, Lee TW, Steenhuis J, Parnot C (2004). Sequential binding of agonists to the beta2 adrenoceptor. Kinetic evidence for intermediate conformational states. J Biol Chem.

[CR53] Swaminath G, Deupi X, Lee TW, Zhu W, Thian FS, Kobilka TS, Kobilka B (2005). Probing the beta2 adrenoceptor binding site with catechol reveals differences in binding and activation by agonists and partial agonists. J Biol Chem.

[CR54] Thomas JP, Roe PL (1993) Development of non-dissipative numerical schemes for computational aeroacoustics. AIAA Paper 93-3382 (AIAA 11th Computational Fluid Dynamics Conference)

[CR55] Toll L, Pajak K, Plazinska A, Jozwiak K, Jimenez L, Kozocas JA, Tanga MJ, Bupp JE, Wainer IW (2012). Thermodynamics and docking of agonists to the β(2)-adrenoceptor determined using [(3)H](R, R′)-4-methoxyfenoterol as the marker ligand. Mol Pharmacol.

[CR56] Torrie GM, Valleau JP (1977). Nonphysical sampling distributions in Monte Carlo free-energy estimation: umbrella sampling. J Comput Phys.

[CR57] van der Spoel D, van Maaren PJ, Berendsen HJC (1998). A systematic study of water models for molecular simulation: derivation of water models optimized for use with a reaction field. J Chem Phys.

[CR58] van Der Spoel D, Lindahl E, Hess B, Groenhof G, Mark AE, Berendsen HJC (2005). Gromacs: fast flexible and free. J Comp Chem.

[CR59] Vassilatis DK, Hohmann JG, Zeng H, Li F, Ranchalis JE, Mortrud MT, Brown A, Rodriguez SS, Weller JR, Wright AC, Bergmann JE, Gaitanaris GA (2003). The G protein-coupled receptor repertoires of human and mouse. Proc Natl Acad Sci USA.

[CR60] Wacker D, Fenalti G, Brown MA, Katritch V, Abagyan R, Cherezov V, Stevens RC (2010). Conserved binding mode of human beta2 adrenergic receptor inverse agonists and antagonist revealed by X-ray crystallography. J Am Chem Soc.

[CR61] Wieland K, Zuurmond HM, Krasel C, Ijzerman AP, Lohse MJ (1996). Involvement of Asn-293 in stereospecific agonist recognition and in activation of the beta 2-adrenergic receptor. Proc Natl Acad Sci U S A.

[CR62] Woo AY, Jozwiak K, Toll L, Tanga MJ, Kozocas JA, Jimenez L, Huang Y, Song Y, Plazinska A, Pajak K, Paul RK, Bernier M, Wainer IW, Xiao RP (2014). Tyrosine 308 is necessary for ligand-directed Gs protein-biased signaling of β2-adrenoceptor. J Biol Chem.

[CR63] Xie KQ, Cao Y, Zhu XZ (2006). Role of the second transmembrane domain of rat adenosine A1 receptor in ligand–receptor interaction. Biochem Pharmacol.

[CR64] Xie H, Cheng Y, Huo Y, Huang G, Su J (2014). Association between β_2_-adrenoceptor gene polymorphisms and asthma risk: an updated meta-analysis. PLoS one.

[CR65] Yao X, Parnot C, Deupi X, Ratnala VR, Swaminath G, Farrens D, Kobilka B (2006). Coupling ligand structure to specific conformational switches in the beta2-adrenoceptor. Nat Chem Biol.

[CR66] Zhang X, Chien EY, Chalmers MJ, Pascal BD, Gatchalian J, Stevens RC, Griffin PR (2010). Dynamics of the beta2-adrenergic G-protein coupled receptor revealed by hydrogen-deuterium exchange. Anal Chem.

[CR67] Zocher M, Fung JJ, Kobilka BK, Müller DJ (2012). Ligand-specific interactions modulate kinetic, energetic, and mechanical properties of the human β_2_ adrenergic receptor. Structure..

