# Supplementary material for: Agonist binding by the β2-adrenergic receptor: an effect of receptor conformation on ligand association–dissociation characteristics
Source: Eur Biophys J. 2015 Mar 1;44(3):149–63. doi: 10.1007/s00249-015-1010-4 (PMC4359354; doi:10.1007/s00249-015-1010-4)
Supplement: Supplementary file 1 — Supplementary material 1 (DOCX 3153 kb) [file 249_2015_1010_MOESM1_ESM.docx]

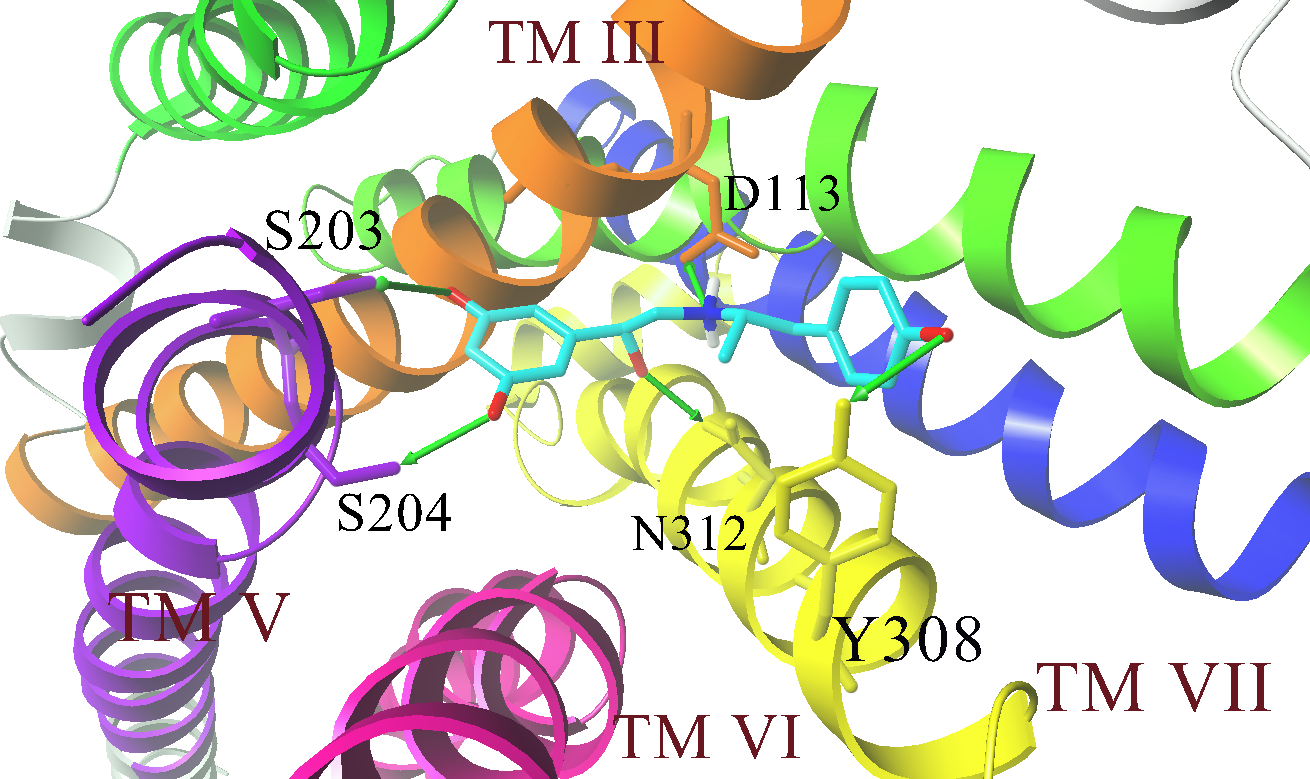


**Fig. S1.** The fenoterol molecule in the complex with In_β_2_-AR. The depicted structure was obtained on the basis of the docking studies described elsewhere [Plazinska et al. 2013]. Some characteristic interactions are represented by green arrows. Hydrogen atoms are omitted for clarity. See the details in the main manuscript.


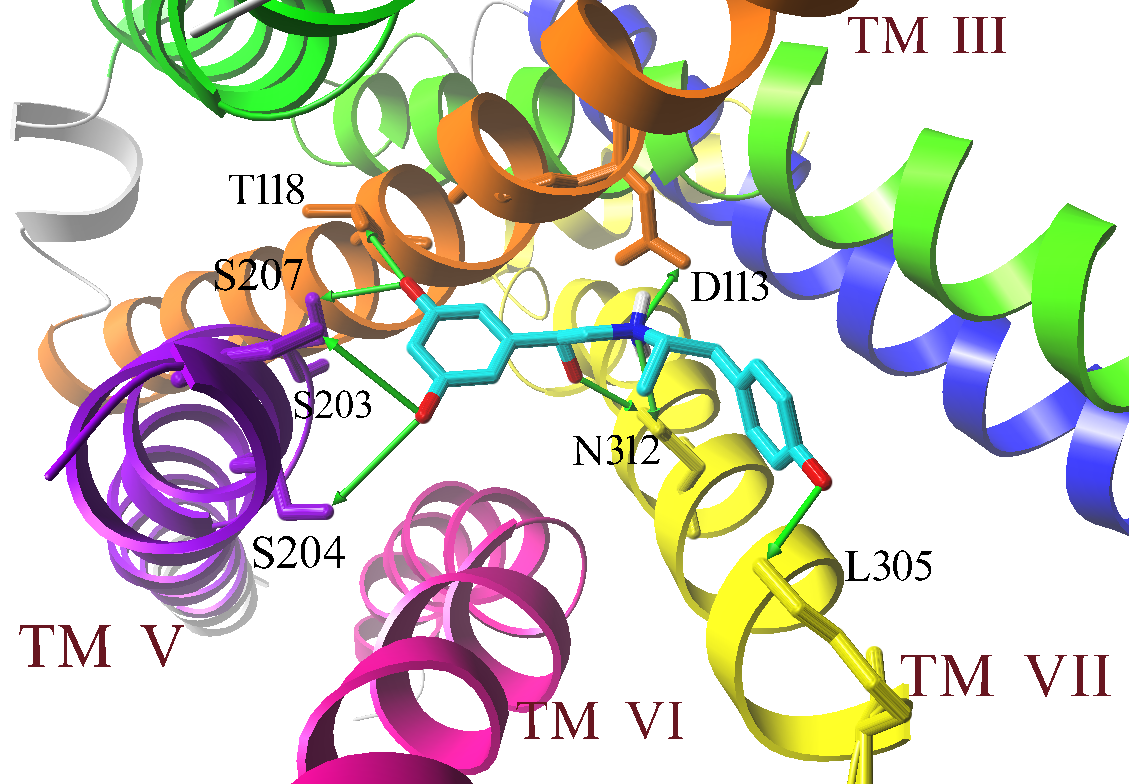


**Fig. S2.** The fenoterol molecule in the complex with Ac_β_2_-AR. The depicted structure was obtained on the basis of the docking studies described elsewhere [Plazinska et al. 2013]. Other details as in Fig. S1.


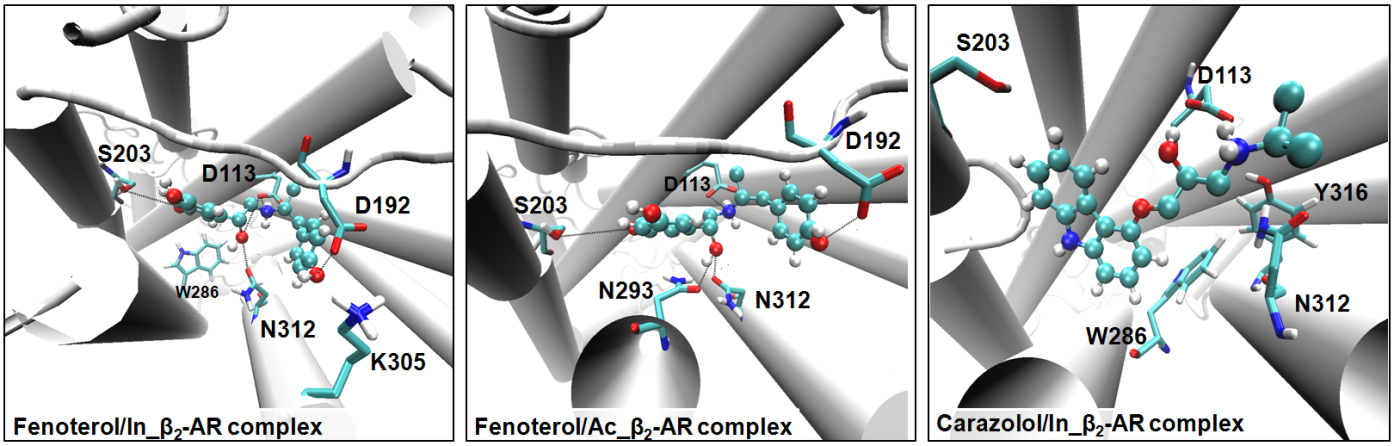


**Fig. SI3**. The final frames of the unconstrained MD runs which were taken for the subsequent ligand-pulling simulations (fragments of the structures depicting the ligand vicinity).


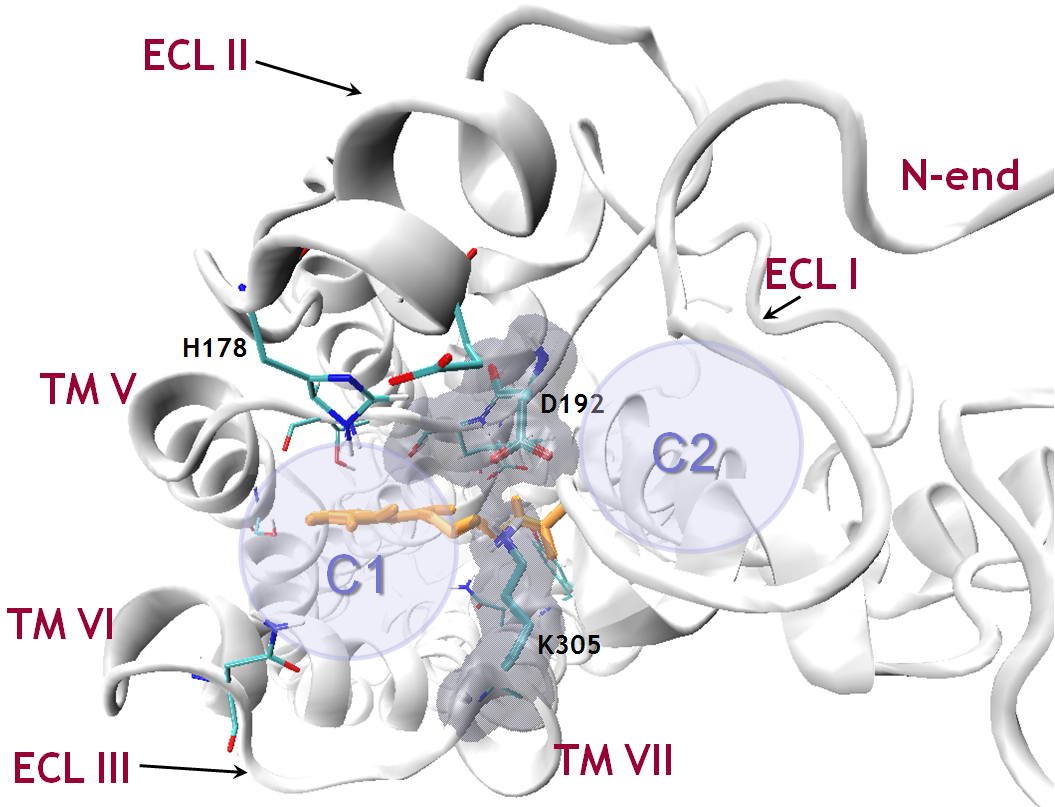


**Fig. SI4.** The fenoterol molecule in the complex with Ac_β_2_-AR (the global minimum of FEP). Circles show the approximate locations of dissociation channels (C1 and C2). Ligand is shown in orange whereas the amino acid side chains within 4 Å from the ligand molecule are shown as sticks and labeled. Solvent-accessible surface of D192 and K305 residues (which separate C1 from C2) is displayed in gray.


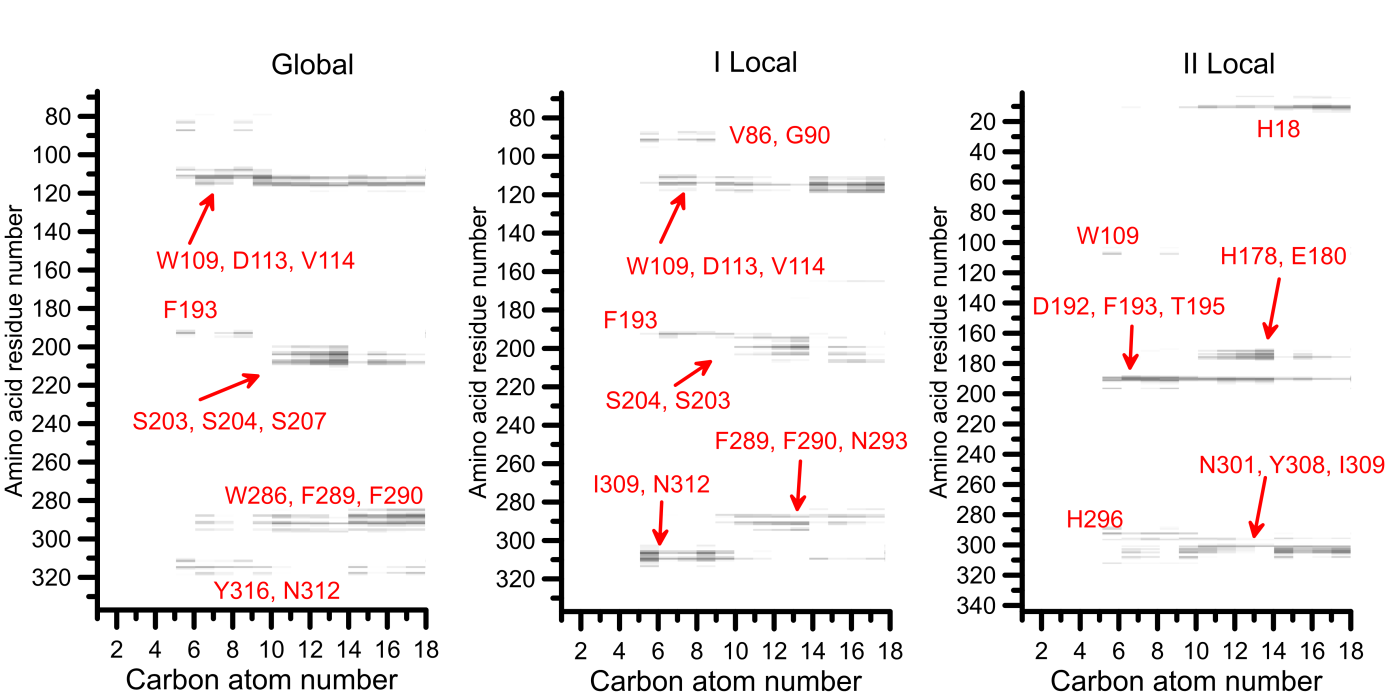


**Fig SI5.** The contact maps generated for the carazolol/In_β_2_-AR complex and the three characteristic points of the FEP curve. The X axis corresponds to the carbon atoms of the ligand molecule whereas the Y axis represents the amino acid residues of β_2_-AR. The most intensive contacts are described in red (see also Tabs. 1 and 2 in the main manuscript). The contact distances are displayed as a color-coded matrix where darker colors indicate residues which are close to given atom of the ligand and lighter colors indicate pairs which are distant from each other. Maps were generated by using the VMD software [Humphrey, W., Dalke, A. and Schulten, K., "VMD - Visual Molecular Dynamics", J. Molec. Graphics, 1996, 14, 33-38].


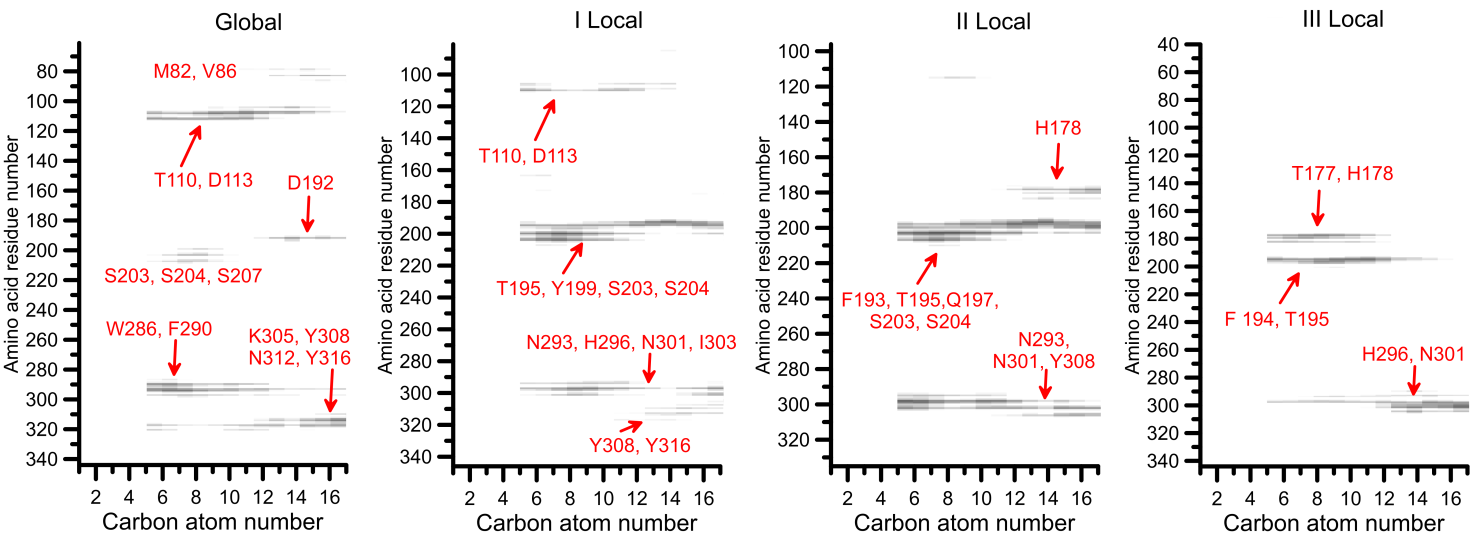


**Fig SI6.** The contact maps generated for the fenoterol/In_β_2_-AR complex and the three characteristic points of the FEP curve. The other details as in Fig. SI4.


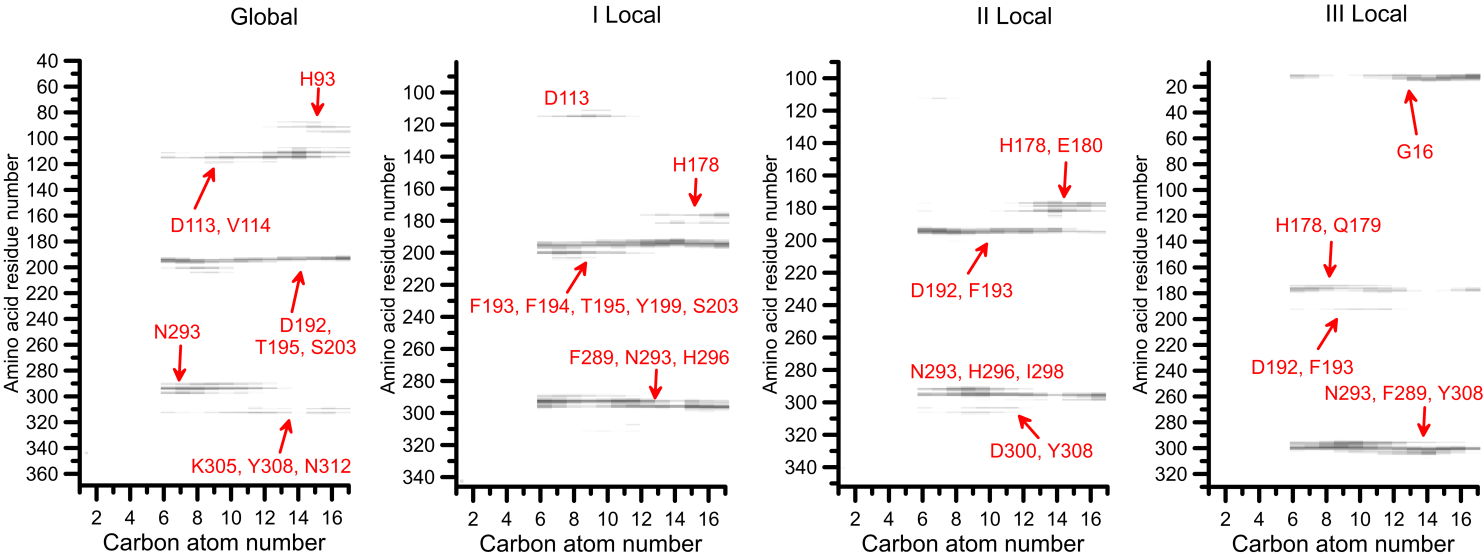


**Fig SI7.** The contact maps generated for the fenoterol/Ac_β_2_-AR complex and the three characteristic points of the FEP curve. The other details as in Fig. SI4.


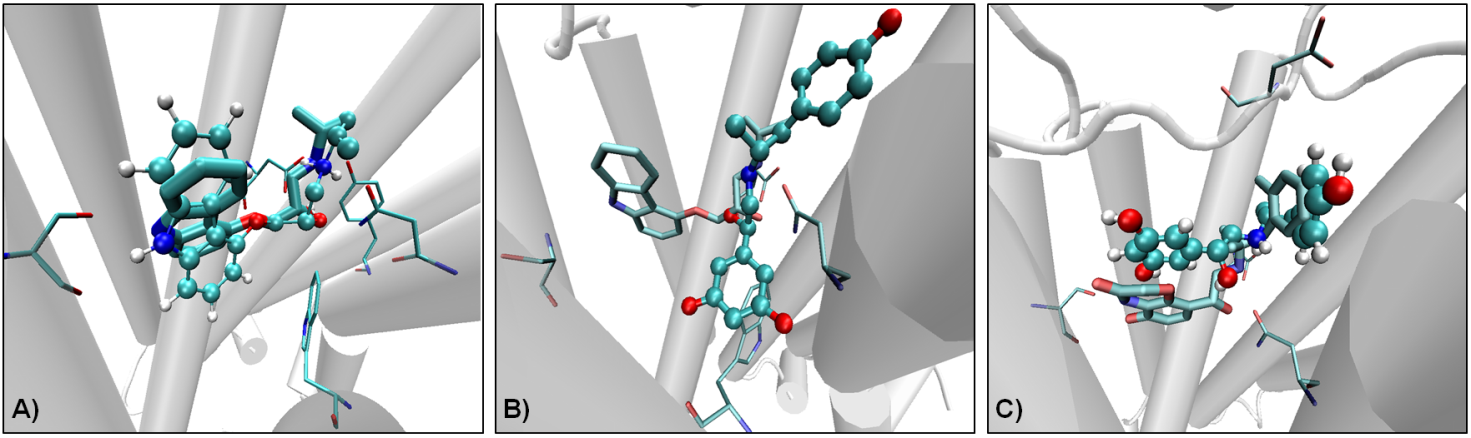


**Fig. SI8.** The comparison of the ligands positions (carazolol (A) and fenoterol (B, C)) in the global minima of FEPs (ball-and-stick) with the positions of the ligands cocrystalized with β_2_-AR: carazolol (PDB: 2RH1 (A, B)) and agonist molecule BI-167107 (PDB:3P0G (C)). The RMSD calculated for all atoms of carazolol with respect to the crystal structure (2RH1) and to the average MD structure (the global FEP minimum) equals to 0.2 Å.
